# Prompt recovery after surgical treatment of pulmonary and aortic valve endocarditis in a patient with acute heart failure

**DOI:** 10.1093/jscr/rjac315

**Published:** 2022-07-04

**Authors:** Aikaterini Gavalaki, George Athanasopoulos, Antonios Roussakis, Michael Koutouzis, Konstantinos Perreas, Ioannis Nenekidis

**Affiliations:** 1st Department of Adult Cardiac Surgery, Onassis Cardiac Surgery Center, Athens, Greece; Cardiology Department, Onassis Cardiac Surgery Centre, Athens, Greece; 1st Department of Adult Cardiac Surgery, Onassis Cardiac Surgery Center, Athens, Greece; Cardiology Department, Red Cross General Hospital, Athens, Greece; 1st Department of Adult Cardiac Surgery, Onassis Cardiac Surgery Center, Athens, Greece; 1st Department of Adult Cardiac Surgery, Onassis Cardiac Surgery Center, Athens, Greece

## Abstract

Infective endocarditis remains a medical challenge among urgent cases of cardiac disease. Multi-valvular endocarditis is uncommon and simultaneous right and left-sided valvular involvement, particularly affecting the pulmonary valve, is scarcely reported. A rare case of a patient with subacute myelodysplastic syndrome, who presented with endocarditis involving both aortic and pulmonary valves, complicated with new-onset heart failure, is described. The patient presented prompt recovery of both right and left ventricular function after combined aortic and pulmonary valve replacement.

## INTRODUCTION

Infective endocarditis (IE) remains a medical challenge among urgent cases of cardiac disease, still carrying a poor prognosis and a high mortality rate reaching 17% even during the first month after hospitalization [1]. Multi-valvular IE is uncommon and simultaneous right and left-sided valvular involvement, particularly the pulmonary valve (PV), is rarely reported.

## CASE REPORT

A 73-year-old male patient was referred to our hospital because of decompensated heart failure, due to both severe aortic and pulmonic valve regurgitation, secondary to endocarditis.

**Figure 1:**
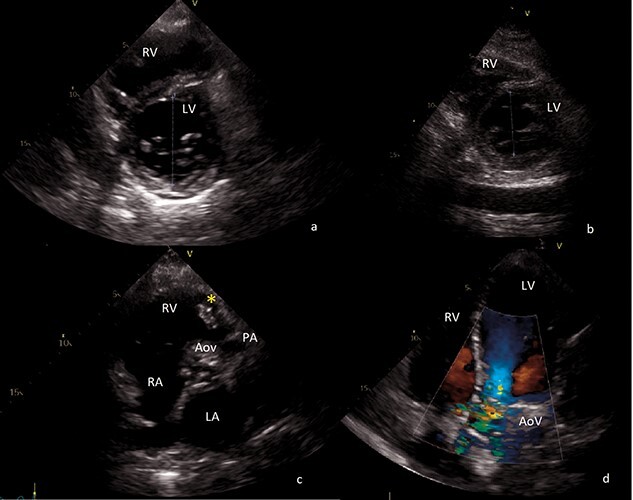
Transthoracic echocardiography images. (**a**) Preoperative short axis view of left ventricle. End-diastolic left ventricle diameter of 68 mm. (**b**) Postoperative short axis view of left ventricle. End-diastolic left ventricle diameter of 43 mm. (**c**) Sizable vegetation (yellow asterisk) of about 4 cm provoking right ventricular outflow obstruction. (**d**) Four-chamber view, showing severe aortic regurgitation. AoV aortic valve; LA left atrium; LV left ventricle; PA pulmonary artery; RA right atrium; RV right ventricle.

Blood cultures initially revealed streptococcus species and the patient was set under antibiotic therapy, till the time he presented to our center. The patient’s medical history included diabetes mellitus type 2, chronic obstructive pulmonary disease, mild chronic renal insufficiency and subacute myelodysplastic syndrome (MDS). Transthoracic echocardiogram revealed:

1. Both right and left ventricle grossly dilated due to excessive volume overload (end-diastolic left ventricle diameter of 68 mm) (Fig. 1a).
2. Severe PV insufficiency, with a single sizable vegetation of about 4 cm provoking right ventricular outflow obstruction (Fig. 1c)
3. Severe aortic valve regurgitation with multiple vegetations, the largest of about 11 mm length, protruding to the aorta as well as to the left ventricle outflow tract (Fig. 1d).

**Figure 2:**
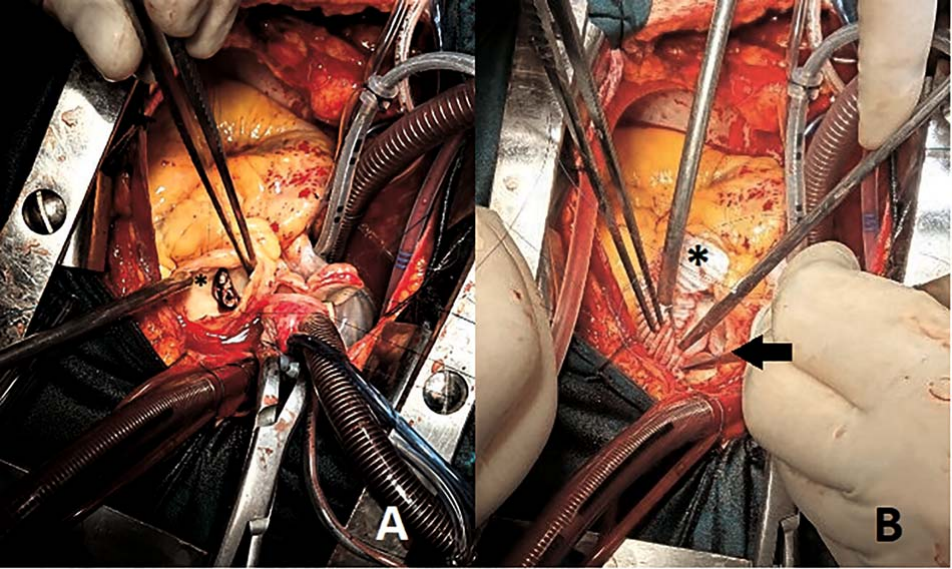
Perioperative images showing (**A**) sizable PV vegetation (asterisk) (**B**) pulmonary bioprosthesis (arrow) and pulmonary artery enlargement using synthetic patch (asterisk).

The patient underwent bivalvular replacement with bioprostheses, following assiduous debridement of aortic and PVs. Pulmonary artery enlargement was essential, using a synthetic patch (Fig. 2). The operation was uneventful and the patient was discharged on the eighth postoperative day.

Before discharge, transthoracic echocardiogram revealed well-functioning bioprostheses, with prompt recovery of both right and left ventricles (Fig. 1b). Native valves’ culture was negative. Follow-up blood tests and echocardiogram at 6 and 12 months after surgery were free of recurrence.

## DISCUSSION

Multi-valvular IE is uncommon and simultaneous right- and left-sided valvular involvement is scarcely reported. PV IE is extremely rare, accounting for 1.5%–2% of total cases of IE [2]. Most data on PV IE are limited to a few minor case series and reports [3].

The most common risk factor for PV endocarditis is intravenous drug abuse. However, multiple other risk factors have been reported including male gender, uncontrolled diabetes, central venous catheters, pacemaker implantation with lead infection, immunosuppression including those with AIDS, organ transplant recipients, and increasingly aggressive therapy for hematologic and solid malignancies, as well as elderly patients with chronic liver disease, chronic renal failure and alcoholics [3, 4].

In our case, among others, MDS is considered to be an important factor. MDS patients commonly have refractory anemia accompanied by various degrees of granulocytopenia and thrombocytopenia. Coagulation disorders, with or without thrombocytopenia, are also common along with an increased tendency of infection, particularly at the valvular replacement site, and transfusional needs of RBCs and platelets [5] during and after open heart surgery. Therefore, the patient remained under close haematologic follow-up, as well as under per os antibiotic therapy for 6 months to avoid prosthetic valve endocarditis recurrence.

As many as 50% of IE patients will require surgery due to persistent bacteremia, emboli or heart failure [1]. Heart failure due to valve dysfunction is the most common indication for surgery; however, timing of surgery is challenging and differs between major European and US guidelines [6, 7]. In the present case, new-onset heart failure due to serious valve regurgitation was the decisive parameter for intervention. In cases with such a clear indication for surgery, referral of intervention concludes to ominous outcome, with < 50% survival for the first month [1].

In native valve IE, acute valvular regurgitation is the most frequent reason of new onset heart failure. Acute severe valvular regurgitation may be caused by leaflet perforation or large mobile vegetations [6, 7]. Vegetations are the hallmark lesions of IE. It is important to identify them early for optimal treatment. Therefore, echocardiography is pivotal to diagnosis and management.

Surgical options include debridement of the infected area, vegetation excision and either valve preservation, valve repair or replacement. In cases, where pulmonic valve replacement is inevitable, the use of a homograft or xenograft is recommended; however, large series of stented bioprosthetic PV replacements has demonstrated favorable results [8].

Our patient’s scenario is unique, since there is limited information regarding patients with MDS, presenting with simultaneous pulmonary and aortic valve IE. Timing of surgical management, as well as post-operative care of these cases, is of particular importance. Moreover, it is of interest for both volume-loaded ventricles showing an early functional recovery postoperatively.
